# Influenza-like illness in cigarette smokers and electronic-cigarette users: a secondary analysis from the PAIVED study

**DOI:** 10.3389/fpubh.2026.1725232

**Published:** 2026-04-16

**Authors:** Rachael D. C. Jones, Kat Schmidt, Christina Schofield, Anuradha Ganesan, Wesley Campbell, David Hrncir, Tahaniyat Lalani, Katrin Mende, Ana E. Markelz, Catherine M. Berjohn, Laurie Housel, Drake H. Tilley, Adam Saperstein, Alan Williams, Bruce McClenathan, Limone Collins, Christina Spooner, Srihari Seshadri, Ryan C. Maves, Robert J. O’Connell, Simon Pollett, Mark P. Simons, John H. Powers, Christian L. Coles, Rhonda E. Colombo, Timothy H. Burgess, Stephanie A. Richard

**Affiliations:** 1 Department of Preventive Medicine, Uniformed Services University of the Health Sciences, Bethesda, MD, United States; 2 Infectious Disease Clinical Research Program, Department of Preventive Medicine and Biostatistics, Uniformed Services University of the Health Sciences, Bethesda, MD, United States; 3 The Henry M. Jackson Foundation for the Advancement of Military Medicine, Inc., Bethesda, MD, United States; 4 Madigan Army Medical Center, Tacoma, WA, United States; 5 Walter Reed National Military Medical Center, Bethesda, MD, United States; 6 Carl R. Darnell Army Medical Center, Fort Hood, TX, United States; 7 Wilford Hall Ambulatory Surgical Center, Lackland Air Force Base, San Antonio, TX, United States; 8 Immunization Healthcare Division, Defense Health Agency, Falls Church, VA, United States; 9 Naval Medical Center Portsmouth, Portsmouth, VA, United States; 10 Brooke Army Medical Center, JBSA Fort Sam Houston, San Houston, TX, United States; 11 Department of Medicine, Uniformed Services University of the Health Sciences, Bethesda, MD, United States; 12 Naval Medical Center San Diego, San Diego, CA, United States; 13 Womack Army Medical Center, Fort Bragg, NC, United States; 14 Navy Medicine Readiness and Training Command, Annapolis, MD, United States; 15 Department of Family Medicine, Uniformed Services University of the Health Sciences, Bethesda, MD, United States; 16 Section of Infectious Diseases, Wake Forest University School of Medicine, Winston-Salem, NC, United States; 17 Clinical Research Directorate, Frederick National Laboratory for Cancer Research, Frederick, MD, United States

**Keywords:** cigarettes, electronic-cigarettes, influenza-like illness, respiratory infections, smoking

## Abstract

**Background:**

The Pragmatic Assessment of Influenza Vaccine Effectiveness in the Department of Defense (PAIVED) study was a clinical trial of three influenza vaccines in military beneficiaries enrolled at 10 military treatment facilities over four influenza seasons (2018/19–2021/22). This secondary analysis aimed to assess the relationship between cigarette smoking and e-cigarette use and influenza-like illness (ILI) incidence and severity.

**Methods:**

Demographic information, including cigarette smoking and e-cigarette use, was collected at enrollment. ILIs were identified during the influenza season of enrollment using weekly surveys. ILI symptoms were reported using the inFLUenza Patient-Reported Outcome (FLU-PRO) instrument. The relationship between smoking status and risk of reporting an ILI was estimated using Poisson regression; ILI severity was compared by cigarette smoking and e-cigarette use using multivariable linear regression.

**Results:**

Among 8,708 participants with cigarette smoking status, 4.3% were current smokers and 11.9% were former smokers. Current cigarette smokers reported higher respiratory domain scores (0.24 (95% CI: 0.07, 0.41)) than non-smokers; former cigarette smokers were at higher risk of reporting an ILI than non-smokers [rate ratio 1.11 (95% CI: 1.02, 1.20)] and reported ILI episodes that were on average 1.23 days longer in duration (95% CI: 0.29, 2.17). Among 8,119 participants with e-cigarette use status, 3.9% were current users and 2.6% were former users. Current e-cigarette users reported more than one additional day with limited activity (1.14 days, 95% CI: 0.10, 2.18) and 0.78 additional days with fever (95% CI, 0.20, 1.35); former e-cigarette users were at higher risk of reporting an ILI than nonusers [rate ratio 1.20 (95% CI, 1.04, 1.38)] and reported higher respiratory domain scores than non-users (0.27 (95% CI 0.08, 0.46)).

**Conclusion:**

We observed slightly higher ILI risk in former cigarette smokers and e-cigarette users, longer symptom duration in former cigarette smokers, and higher respiratory symptom scores in current cigarette smokers and former e-cigarette users compared to non-smokers/users. Findings in this secondary analysis are exploratory and hypothesis-generating; additional studies are needed to confirm the relationships reported here.

## Highlights

- This analysis examined the link between cigarette smoking/e-cigarette use and influenza-like illness (ILI) in military beneficiaries.
- Former cigarette smokers and former e-cigarette users were at higher risk of ILI.
- Current cigarette smokers and former e-cigarette users reported more severe respiratory symptoms than non-smokers/users.

## Introduction

Influenza and influenza-like illness (ILI) are associated with significant morbidity and mortality in the United States (US). During the 2023–2024 season, influenza caused 470,000 hospitalizations and 28,000 deaths in the US (1). In 2023, among US military service members, respiratory infections were responsible for 2.5% of outpatient clinic visits despite high vaccination rates (2), and respiratory infections can be a major problem in military training and deployment environments due to high stress levels and crowded conditions (3).

Previous studies have reported that cigarette smokers were at increased risk of developing an ILI (4, 5). A systematic review found that cigarette smokers were more likely to report an ILI and to be diagnosed with influenza than non-smokers (6). Another systematic review and meta-analysis demonstrated that individuals with influenza infections who smoked cigarettes had significantly higher odds of hospitalization and intensive care unit admission compared to non-smokers (7). Cigarette smoke is thought to injure the respiratory defense mechanisms and impair immune function, impacting macrophages, neutrophils, and airway surface liquid in the lungs (8, 9).

While e-cigarettes have been marketed as a healthier alternative to cigarettes, they can also impact immune function and increase inflammation with long-term health impacts (10–14). Cigarettes contain harmful chemical additives that impact immune function by creating prolonged inflammatory responses that cause structural damage to airways (15). E-cigarettes share similar components with cigarettes; in addition, they also possess toxic chemicals that can contribute to more severe ILI (16). E-cigarettes are thought to decrease immune response in the nasal mucosa of the respiratory tract, reduce cytokines that regulate antiviral host defense responses, and impact macrophages, neutrophils, and airway surface liquid functions (17).

Prior studies have primarily reported on the effects of cigarettes on influenza risk and severity (6–9, 18, 19); data on the relationship between cigarette smoking and ILI symptom severity has been less fully explored. In addition, the relationship between e-cigarette use and ILI risk and severity remains limited. The objective of this study is to evaluate the incidence and severity of ILI among cigarette smokers and e-cigarette users compared with non-smokers and non-users in a relatively young, healthy, and influenza-vaccinated population, with the aim of providing meaningful insights into this public health issue.

## Methods

### Study design

The Pragmatic Assessment of Influenza Vaccine Effectiveness in the DoD (PAIVED) study was a pragmatic randomized clinical trial comparing the effectiveness of licensed egg-based inactivated, cell-culture-based inactivated, and recombinant influenza vaccine (20). Participants were enrolled, vaccinated, and followed through an influenza season (2018/19, 2019/20, 2020/21, and 2021/22) for the development of influenza and/or ILI. We leveraged this trial to perform a secondary analysis of the association between cigarette smoking/e-cigarette use and ILI risk and severity. The overall PAIVED study results showed no statistically or clinically significant differences in ILI incidence between the egg-based and non-egg-based vaccines, allowing a pooled analysis of participants across the three vaccines.

### Study population

Adult participants aged 18 years or older who were eligible for care in the US Military Health System (MHS) were voluntarily enrolled in PAIVED during influenza vaccination events at 10 military treatment facilities across the US (the number of sites varied by season). Individuals were excluded from the study if they had already received an influenza vaccine for the current season, intended to receive another influenza vaccine type, or were unable to receive a standard dose influenza vaccine due to a medical condition or allergy.

### Study variables

The exposures of interest for this analysis were cigarette smoking and e-cigarette use status; e-cigarette use was added to the demographic form in the second season (2019/20) of the study and therefore was not available for the first PAIVED season. The enrollment form also included questions about the number of years the participant smoked cigarettes or used e-cigarettes, and number of times the participants smoked cigarettes or used e-cigarettes in the 7 days prior to enrollment. Other variables were collected at enrollment (e.g., sex, age, height, weight, education history, military status, healthcare worker status), and body mass index (BMI) was calculated as weight (kg)/height (m^2^). History of chronic pulmonary disease was identified in the participant’s medical record in the year prior to study entry and included in a sensitivity analysis.

The outcomes of interest were ILI incidence, symptom severity, and duration of symptoms. ILIs were identified using weekly surveys sent to participants and through healthcare encounter data from the MHS Data Repository (MDR). Participants with study-identified ILIs completed an acute visit with study staff upon notification of an ILI, and a convalescent visit 21 days later. During those visits, information was collected about the ILI, such as the number of days ill, days with limited activity, and days with fever. ILI severity was assessed using the InFLUenza Patient-Reported Outcome (FLU-PRO) survey which was sent to the participant daily for seven days. The FLU-PRO instrument is a standardized and validated method to quantify ILI severity and recovery (21, 22) and includes questions on symptom severity in six specific body systems (nose, throat, eye, respiratory, gastrointestinal, and systemic). Symptom severity was captured using 5-point Likert scale items that ranged from 0 (“Not at all”) to 4 (“Very much”). Mean symptom scores within domains and overall were generated, and the maximum values were used in the analyses.

### Statistical analysis

Pearson’s Chi-squared and Kruskal–Wallis tests were used to compare selected characteristics and baseline demographics among cigarette smoking and e-cigarette use categories. Although global comparisons did not specify which pairs differed significantly, multivariable analysis was used to further evaluate these associations. Poisson regression was used to compare the risk of reporting ILI by cigarette smoking/e-cigarette use status, controlling for sex, age, BMI, the season of enrollment, education, military status, healthcare worker status, and the percentage of ILI surveys completed. In addition, history of chronic pulmonary disease was included in the multivariable model as a sensitivity analysis. Among participants who reported an ILI, multivariable linear regression was used to compare ILI symptom duration and severity by cigarette smoking and e-cigarette use status, controlling for sex, age, BMI, the season of enrollment, education history, military status, and healthcare worker status. A sensitivity analysis was performed in a subset of participants who responded to at least 75% of the weekly surveys.

Participants were excluded from the analysis based on incomplete or missing data (<50% response rate to ILI surveys, incomplete covariate data, missing cigarette smoking or e-cigarette use data). Cigarette smoking and e-cigarette use were analyzed separately. Sensitivity analyses were performed using cigarette smoking/e-cigarette use frequency and duration, and by combining cigarette smoking and e-cigarette use into a single ‘smoking’ category. Analyses were performed using R (R Version 4.4.0, R Core Team 2021). *p*-value < 0.05 was used to determine statistical significance.

### Ethical approval

This study was conducted following Good Clinical Practice guidelines and according to the Declaration of Helsinki guidelines. PAIVED (IDCRP-120) was approved by the Uniformed Services University Institutional Review Board. All study participants provided written informed consent prior to enrollment. The PAIVED study is registered at ClinicalTrials.gov (identifier NCT03734237).

## Results

**Figure 1:**
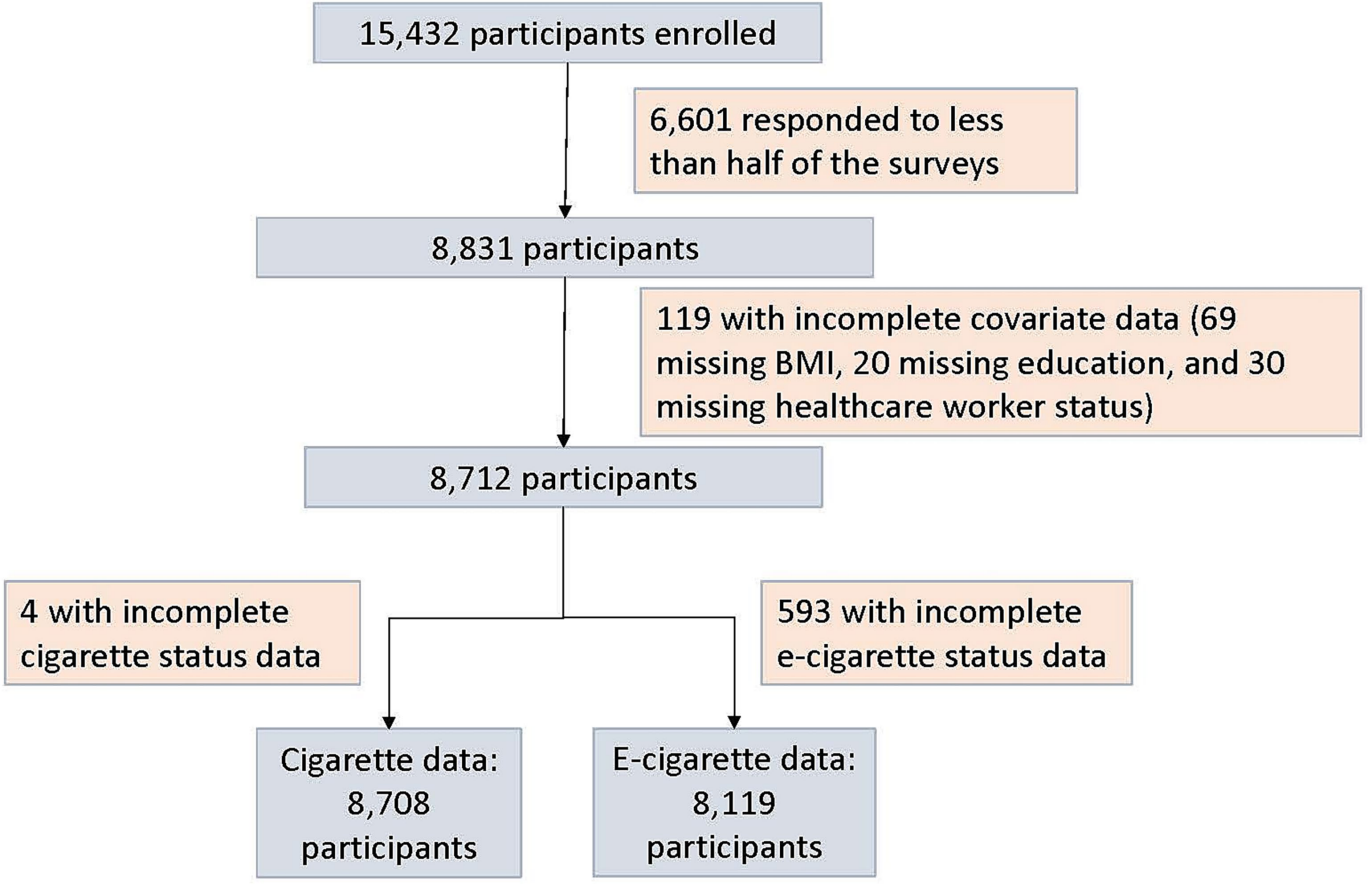
Flowchart of PAIVED participants included in analysis.

Among 15,432 participants enrolled and vaccinated in PAIVED, 8,708 participants were retained in the cigarette smoking analysis and 8,119 participants were retained in the e-cigarette use analysis (Figure 1). Most participants reported that they did not smoke cigarettes (83.7%) or use e-cigarettes (93.5%) (Table 1). Current cigarette smokers and current e-cigarette users tended to be male, had less formal education, and responded to fewer weekly surveys than participants who reported no cigarette smoking or e-cigarette use. Current e-cigarette users also tended to be younger than nonusers. Among those with both cigarette and e-cigarette use data, few reported both smoking cigarettes and using e-cigarettes (56/8,117, 0.69%). Cigarette use and e-cigarette use were analyzed separately; combined analysis of those reporting current or historical use of both cigarettes and e-cigarettes was not possible based on small numbers.

**Table 1:** Characteristics and influenza-like illness outcomes of PAIVED participants with cigarette smoking status data (*N* = 8,708) and e-cigarette smoking status data (*N* = 8,119).

Characteristics	Cigarette smoking status				E-cigarette use status			
	Current (*N* = 378)	Former (*N* = 1,039)	Never (*N* = 7,291)	*p*-value	Current (*N* = 315)	Former (*N* = 212)	Never (*N* = 7,592)	*p*-value
Female^a^	99 (26.2%)	350 (33.7%)	3,053 (41.9%)	<0.01	93 (29.5%)	63 (29.7%)	3,056 (40.3%)	<0.01
Age, mean (SD)^b^	39.9 (14.2)	45.4 (15.3)	38.0 (13.9)	<0.01	28.8 (9.3)	30.9 (9.0)	38.6 (13.8)	<0.01
BMI, mean (SD)^b^	27.6 (5.0)	29.2 (5.1)	27.0 (4.7)	<0.01	26.9 (4.3)	27.7 (4.3)	27.2 (4.7)	0.05
Influenza season^a^				<0.01				<0.01
2018–2019	31 (8.2%)	124 (11.9%)	424 (5.8%)		–	–	–	
2019–2020	151 (39.9%)	376 (36.2%)	2,559 (35.1%)		87 (27.6%)	56 (26.4%)	2,943 (38.8%)	
2020–2021	98 (25.9%)	218 (21.0%)	1,650 (22.6%)		88 (27.9%)	56 (26.4%)	1822 (24.0%)	
2021–2022	98 (25.9%)	321 (30.9%)	2,658 (36.5%)		140 (44.4%)	100 (47.2%)	2,827 (37.2%)	
Education^a^				<0.01				<0.01
High school or less	168 (44.4%)	286 (27.5%)	1,850 (25.4%)		203 (64.4%)	105 (49.5%)	1,873 (24.7%)	
Associate/bachelor’s	160 (42.3%)	504 (48.5%)	2,557 (35.1%)		96 (30.5%)	81 (38.2%)	2,777 (36.6%)	
Master’s/PhD	50 (13.2%)	249 (24.0%)	2,884 (39.6%)		16 (5.1%)	26 (12.3%)	2,942 (38.8%)	
Military status^a^				<0.01				<0.01
Active duty	237 (62.7%)	545 (52.5%)	5,209 (71.4%)		285 (90.5%)	186 (87.7%)	5,405 (71.2%)	
Dependent	47 (12.4%)	181 (17.4%)	1,019 (14.0%)		18 (5.7%)	17 (8.0%)	986 (13.0%)	
Retired military	94 (24.9%)	313 (30.1%)	1,063 (14.6%)		12 (3.8%)	9 (4.2%)	1,201 (15.8%)	
Healthcare worker^a^	79 (20.9%)	289 (27.8%)	3,054 (41.9%)	<0.01	93 (29.5%)	67 (31.6%)	3,209 (42.3%)	<0.01
% of weekly surveys with response, mean (SD)^b^	87.1 (14.9)	90.4 (13.6)	90.5 (13.6)	<0.01	84.0 (16.0)	87.4 (15.1)	90.6 (13.5)	<0.01
ILI reported^a^	129 (34.1%)	413 (39.7%)	2,713 (37.2%)	0.12	124 (39.4%)	100 (47.2%)	2,856 (37.6%)	0.02
Duration, mean (SD)^b^^,^ ^c^	12.5 (7.3)	13.4 (8.8)	11.8 (7.7)	<0.01	11.9 (7.4)	13.0 (7.4)	11.7 (7.2)	0.22
Days with limited activity, mean (SD)^b^^,^ ^d^	5.8 (5.7)	6.1 (6.0)	5.2 (5.8)	0.02	5.5 (5.1)	5.8 (5.4)	5.1 (5.1)	0.41
Days with fever, mean (SD)^b^^,^ ^d^	2.8 (2.3)	3.0 (3.2)	2.5 (3.0)	<0.01	3.1 (3.3)	2.8 (3.2)	2.4 (2.7)	0.07

a Pearson’s chi-square tests.

b Kruskal–Wallis.

c *N* = 2,367 (cigarettes)/*N* = 2,239 (e-cigarettes).

d *N* = 2,346 (cigarettes)/*N* = 2,218 (e-cigarettes).

Values are number (%) unless otherwise specified. SD, standard deviation; BMI, body mass index; ILI, influenza-like illness.

ILI incidence was similar among current, former, and never cigarette smokers (34.1, 39.7, and 37.2%, respectively; *p* = 0.12), whereas the mean ILI duration was different among the groups (12.5, 13.4, and 11.8 days, respectively; *p* < 0.01), as were the number of days with limited activity and fever (Table 1). The only statistically significant difference by e-cigarette use status was in reporting an ILI; 39.4% of current e-cigarette users, 47.2% of former e-cigarette users, and 37.6% of nonusers reported an ILI (*p* = 0.02). Other ILI characteristics such as days with limited activity or fever were not statistically significantly different by e-cigarette use status using bivariable analysis.

In multivariable analysis, former cigarette smokers and former e-cigarette users were more likely to report ILI (1.11, 95% CI: 1.02, 1.20; 1.20, 95% CI: 1.04, 1.38, respectively), compared to non-smokers/non-users after adjusting for sex, age, BMI, influenza season, education, military status, healthcare worker status, and responsiveness to weekly surveys (Supplementary Table 1). No differences were observed in risk of ILI between current cigarette smokers and non-smokers or current e-cigarette users and non-users. Sensitivity analyses were performed that produced very similar results for smoking status and e-cigarette use; e.g., in a subset of participants who responded to at least 75% of the weekly surveys (Supplementary Table 2) and in a model that also included history of chronic pulmonary disease (Supplementary Table 3).

**Figure 2:**
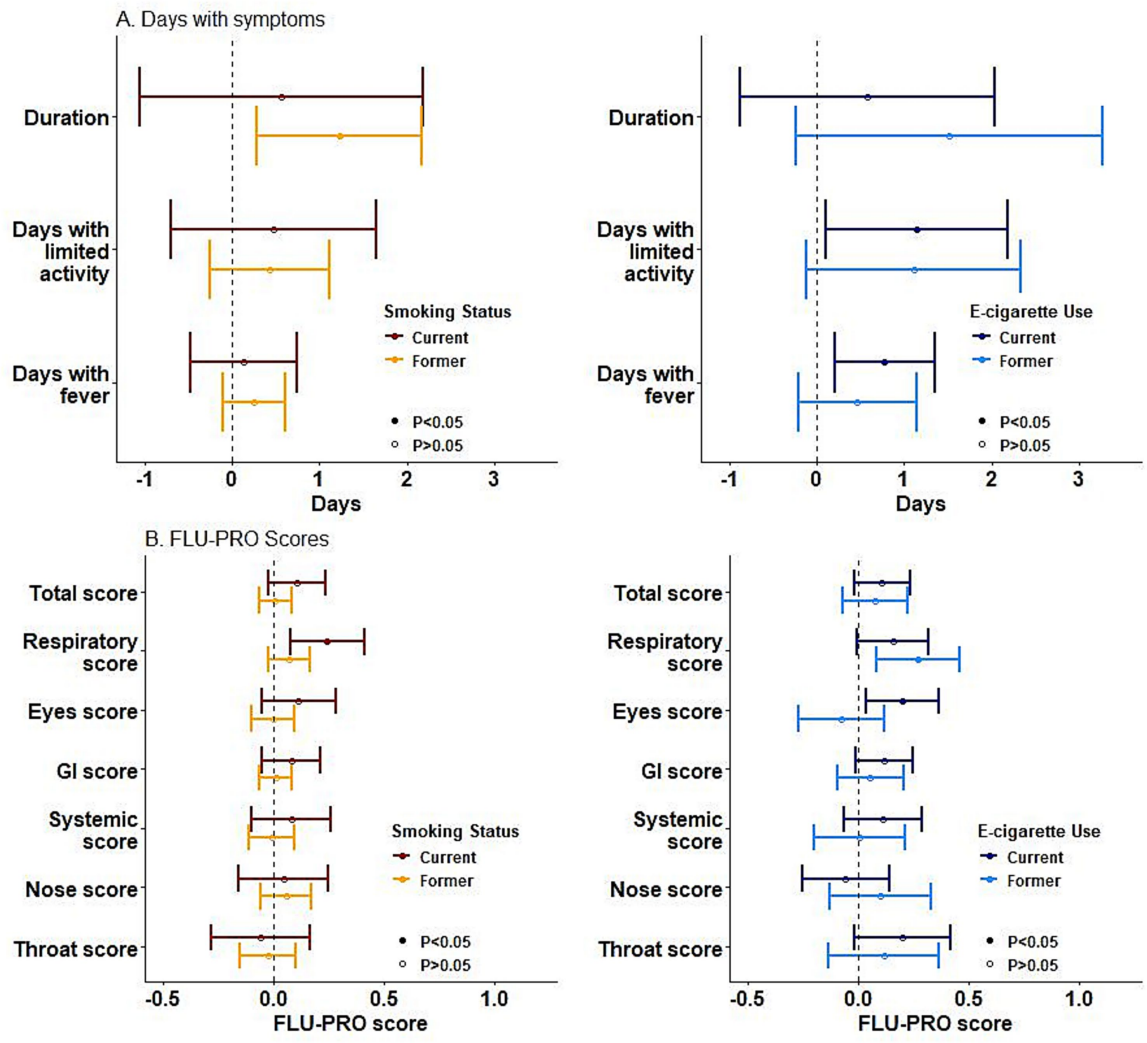
Multivariable linear regression used to test if smoking status (cigarette use)/e-cigarette use significantly predicted ILI severity outcomes, controlling for sex, age, BMI, influenza season, education level, healthcare worker status, and military status.

Former cigarette smokers reported ILI episodes that were on average 1.23 days longer in duration than nonsmokers (95% CI: 0.29, 2.17) (Figure 2) in multivariable models; current e-cigarette users reported 1.14 more days with limited activity (95% CI: 0.10, 2.18) and 0.78 more days with fever (95% CI: 0.20, 1.35) compared to non-users. With respect to FLU-PRO scores, the number of FLU-PRO surveys completed did not differ by smoking/e-cigarette use status (cigarettes: mean surveys = 5.3, 5.3, and 5.1 for current, former, and non-smokers, *p* = 0.35; e-cigarettes: mean surveys = 5.0, 5.2, and 5.2 for current, former, and non-users, *p* = 0.45). Current cigarette smokers and former e-cigarette users had higher maximum respiratory domain scores than non-smokers/non-users (current cigarette smokers: 0.24, 95% CI: 0.07, 0.41; former e-cigarette users: 0.27, 95% CI: 0.08, 0.46) (Supplementary Table 4, Figure 2). Additionally, current e-cigarette users had higher eye domain scores (0.20, 95% CI: 0.03, 0.36). No differences were observed for cigarette smoking or e-cigarette use status for total FLU-PRO scores, or for nose, throat, gastrointestinal, or systemic domain scores. Sensitivity analyses that incorporated smoking frequency and smoking history produced similar findings to those reported above (Supplementary Figures 1–3).

## Discussion

Cigarette smoking and e-cigarette use, both past and present, had variable impacts on the incidence, duration, and severity of ILI. Former cigarette smokers and e-cigarette users were more likely to report ILI compared to non-smokers/non-users, whereas no difference was observed for current cigarette smokers and e-cigarette users. Among those participants who reported an ILI, we found former cigarette smokers reported longer episode duration and current e-cigarette users reported more days with fever and limited activity than did non-smokers/non-users. Maximum FLU-PRO scores did not differ by cigarette smoking or e-cigarette user status, with two exceptions; current cigarette smokers and former e-cigarette users reported more severe respiratory symptom scores, while current e-cigarette users reported more severe eye symptom scores.

Previous studies identified a relationship between current cigarette smoking and ILI or influenza risk (4–6, 19) which was not found in this analysis; several factors may have contributed to the lack of association observed in this analysis. Smoking was reported less frequently by PAIVED participants than has been reported in other populations (5, 19), including active-duty military (23). Potential explanations for this low smoking prevalence include a younger population (cohort effect), and a concern about having smoking status or e-cigarette use documented in the military record (for the active-duty military segment of this sample). These lower current smoking rates may have impacted our ability to detect a difference between current cigarette smokers or e-cigarette users and non-smokers/non-users. Other differences in this population include that PAIVED participants were primarily young, healthy, male active-duty service members who were all vaccinated against influenza, whereas participants in other studies tended to be older, with more females, and lower influenza vaccination rates.

Other studies observed a relationship between cigarette smoking and ILI/influenza-associated risk of hospitalization (7, 18), but the relationship between cigarette smoking and outpatient ILI symptom severity—particularly in a predominately young and healthy population—has been less fully explored. Aside from respiratory scores, current or former cigarette smokers did not report more severe symptoms in other domains. However, with respect to increased ILI symptom duration, former cigarette smokers reported ILI episodes that were longer in duration, even after controlling for age and other factors. This may be due to comorbidities, like chronic airway disease and airway-associated or systemic immune function; further research is warranted.

Some strengths of this study include the large sample size, all of whom were vaccinated against influenza, with prospectively collected ILI data using active surveillance. In addition, this study evaluated not only cigarette smoking, but also e-cigarette use, and the association with ILI risk and severity in a young, healthy population. Limitations of this study include the potential misclassification of smoking status due to disclosure concerns, changes in smoking status from baseline to the time of the ILI, and the potential for reverse causality, whereby individuals quit smoking or using e-cigarettes due to incident health issues; all of these limitations would decrease our ability to detect a difference among the smoking groups. In addition, survey response rate differences between smokers and nonsmokers may have led to differential identification of ILIs; however, sub-analyses indicate that response rate is not associated with reporting an ILI [mean % response 90.4% (no ILI) vs. 90.1% (ILI), *p* = 0.32], and a sensitivity analysis in high responders (75%+) produced very similar results (Supplementary Table 2). The higher ILI incidence among former smokers and e-cigarette users and not current smokers/users merits further study, including exploration of the potential for reverse causality and misclassification. Another potential limitation of this study is that it is a secondary analysis, and thus the results may be best interpreted as exploratory and hypothesis-generating. Finally, other potential co-variates that could be clinical confounders, like asthma and other co-morbidities, were not adjusted for in this analysis.

Tobacco control and regulation are useful tools to decrease tobacco sales and consumption; such tools may prove beneficial with e-cigarettes, as much remains unclear about the long-term health effects of these alternatives to traditional cigarettes (10, 12, 24). There are many potential future directions where additional research is needed, such as identifying the consequences of e-cigarette use on other health conditions, and if found, describing mechanisms and pathways associated with impaired immunity and/or respiratory symptoms. Research into the implications of a dose–response relationship between the number of cigarettes smoked or the number of times e-cigarettes are used per day may also help us further understand their relationship with respiratory symptom severity.

Overall, this study showed small differences between cigarette smokers and e-cigarette users and non-smokers/non-users in ILI and symptom severity, some of which, although the differences are statistically significant, might not reach clinical significance. However, the prevalence of ILI and increasing popularity of e-cigarettes in young, otherwise healthy populations may result in substantive public health impacts. Further studies are needed to evaluate the short- and long-term impact of e-cigarettes on ILI as an emerging public health issue.

## Data Availability

The data analyzed in this study is subject to the following licenses/restrictions: data for this study are held by the Infectious Disease Clinical Research Program (IDCRP), headquartered at the Uniformed Services University of the Health Sciences (USU), Department of Preventive Medicine and Biostatistics. Restrictions apply to the availability of these data, which were used under federal Data User Agreements for the current study and so are not publicly available. Upon request, limited data set(s) may be available, subject to regulatory review and approval. Requests to access these datasets should be directed to contactus@idcrp.org. Full protocol accessible at: https://clinicaltrials.gov/study/NCT03734237.

## Author disclaimer

The views expressed are those of the authors and do not reflect the official policy of the Department of the Army, Department of the Navy, the Department of the Air Force, the Department of War or the US Government, the US Army Medical Dept, US Army Office of the SG, BAMC, MAMC, WRNMMC, WAMC, the Henry M. Jackson Foundation for the Advancement of Military Medicine, Inc. (HJF), Uniformed Services University of the Health Sciences (USUHS), Defense Health Agency, or the US Naval Academy. Mention of trade names, commercial products, or organizations does not imply endorsement by the US Government. Investigators followed human subjects’ protection 45CFR46 policies. The investigators have adhered to the policies for protection of human subjects as prescribed in 45 CFR 46.
