# Supplementary material for: Influenza-like illness in cigarette smokers and electronic-cigarette users: a secondary analysis from the PAIVED study
Source: Front Public Health. 2026 Apr 16;14:1725232. doi: 10.3389/fpubh.2026.1725232 (PMC13130645; doi:10.3389/fpubh.2026.1725232)

**Supplemental materials**

Supplemental Table 1. Influenza-like illness (ILI) incidence rate ratios calculated using Poisson regression in two datasets, one includes cigarette smoking status as a contributing factor (N=8,708) and the other includes electronic cigarette user status (N=8,119). Multivariable models included all variables listed in the table. Models adjusted for sex, age, BMI, influenza season, education, military status, healthcare worker, and percent response to weekly surveillance surveys.

|  | Cigarette smoking status  Rate Ratio (95% CI) | E-cigarette use status  Rate Ratio (95% CI) |
| --- | --- | --- |
| Smoking/User Status  (Nonsmoker/Nonuser) | 1.0 | 1.0 |
| Current Smoker/User | 0.99 (0.86, 1.14) | 1.00 (0.87, 1.15) |
| Former Smoker/User | 1.11 (1.02, 1.20) | 1.20 (1.04, 1.38) |
| Sex  (Female) | 1.0 | 1.0 |
| Male | 0.79 (0.74, 0.83) | 0.79 (0.74, 0.84) |
| Age | 1.00 (0.99, 1.00) | 1.00 (0.99, 1.00) |
| BMI | 1.02 (1.01, 1.03) | 1.02 (1.01, 1.03) |
| Season ^a^  (2018-2019) | 1.0 |  |
| 2019-2020 | 1.26 (1.10, 1.44) | 1.0 |
| 2020-2021 | 0.68 (0.58, 0.79) | 0.54 (0.49, 0.59) |
| 2021-2022 | 1.37 (1.20, 1.57) | 1.09 (1.03, 1.16) |
| Education  (High school or less) | 1.0 | 1.0 |
| Associate or Bachelor | 1.03 (0.96, 1.11) | 1.03 (0.96, 1.11) |
| PhD or Master’s | 0.93 (0.86, 1.01) | 0.93 (0.85, 1.01) |
| Military Status  (Active duty) | 1.0 | 1.0 |
| Retired military | 0.87 (0.78, 0.97) | 0.88 (0.79, 0.99) |
| Dependent | 0.85 (0.77, 0.93) | 0.86 (0.77, 0.95) |
| Healthcare  (No) | 1.0 | 1.0 |
| Yes | 1.10 (1.04, 1.17) | 1.10 (1.03, 1.17) |
| Percent surveillance response | 1.00 (0.99, 1.00) | 1.00 (1.00, 1.00) |
| Smoking Status = Cigarettes; User Status = E-cigarettes   1. e-cigarette data was not collected in the 2018-2019 season | | |

Supplemental Table 2. Sensitivity analysis including only those with 75%+ response to weekly surveys; Influenza-like illness (ILI) incidence rate ratios calculated using Poisson regression in two datasets, one includes cigarette smoking status as a contributing factor (N=7,372) and the other includes electronic cigarette user status (N=6,864). Multivariable models included all variables listed in the table. Models adjusted for sex, age, BMI, influenza season, education, military status, healthcare worker, and percent response to weekly surveillance surveys.

|  | Cigarette smoking status  Rate Ratio (95% CI) | E-cigarette use status  Rate Ratio (95% CI) |
| --- | --- | --- |
| Smoking/User Status  (Nonsmoker/Nonuser) | 1.0 | 1.0 |
| Current Smoker/User | 0.98 (0.83, 1.15) | 0.95 (0.81, 1.13) |
| Former Smoker/User | 1.12 (1.03, 1.22) | 1.20 (1.03, 1.41) |
| Sex  (Female) | 1.0 | 1.0 |
| Male | 0.80 (0.75, 0.85) | 0.80 (0.75, 0.85) |
| Age | 1.00 (0.99, 1.00) | 1.00 (0.99, 1.00) |
| BMI | 1.02 (1.01, 1.03) | 1.02 (1.01, 1.03) |
| Season ^a^  (2018-2019) | 1.0 |  |
| 2019-2020 | 1.22 (1.06, 1.41) | 1.0 |
| 2020-2021 | 0.67 (0.56, 0.79) | 0.54 (0.49, 0.60) |
| 2021-2022 | 1.39 (1.20, 1.60) | 1.13 (1.06, 1.21) |
| Education  (High school or less) | 1.0 | 1.0 |
| Associate or Bachelor | 1.04 (0.96, 1.12) | 1.03 (0.95, 1.12) |
| PhD or Master’s | 0.94 (0.86, 1.03) | 0.93 (0.85, 1.02) |
| Military Status  (Active duty) | 1.0 | 1.0 |
| Retired military | 0.88 (0.78, 0.99) | 0.89 (0.79, 1.01) |
| Dependent | 0.87 (0.78, 0.96) | 0.87 (0.78, 0.97) |
| Healthcare  (No) | 1.0 | 1.0 |
| Yes | 1.09 (1.02, 1.17) | 1.09 (1.02, 1.17) |
| Percent surveillance response | 0.99 (0.99, 1.00) | 0.99 (0.99, 1.00) |
| Smoking Status = Cigarettes; User Status = E-cigarettes   1. e-cigarette data was not collected in the 2018-2019 season | | |

Supplemental Table 3. Sensitivity analysis including history of chronic pulmonary disease in the model; Influenza-like illness (ILI) incidence rate ratios calculated using Poisson regression in two datasets, one includes cigarette smoking status as a contributing factor (N=8,708) and the other includes electronic cigarette user status (N=8,119). Multivariable models included all variables listed in the table. Models adjusted for sex, age, BMI, influenza season, education, military status, history of chronic pulmonary disease in the past year, healthcare worker, and percent response to weekly surveillance surveys. History of chronic pulmonary disease (N=318) was identified in the medical record using the following ICD-10 codes: 27.8, I27.9, J40.x–J47.x, J60.x–J67.x, J68.4, J70.1, J70.3.

|  | Cigarette smoking status  Rate Ratio (95% CI) | E-cigarette use status  Rate Ratio (95% CI) |
| --- | --- | --- |
| Smoking/User Status  (Nonsmoker/Nonuser) | 1.0 | 1.0 |
| Current Smoker/User | 0.97 (0.84, 1.12) | 0.98 (0.85, 1.13) |
| Former Smoker/User | 1.11 (1.02, 1.20) | 1.20 (1.04, 1.38) |
| Sex  (Female) | 1.0 | 1.0 |
| Male | 0.79 (0.74, 0.84) | 0.79 (0.75, 0.84) |
| Age | 1.00 (0.99, 1.00) | 1.00 (0.99, 1.00) |
| BMI | 1.02 (1.01, 1.03) | 1.02 (1.01, 1.03) |
| Season ^a^  (2018-2019) | 1.0 |  |
| 2019-2020 | 1.26 (1.10, 1.44) | 1.0 |
| 2020-2021 | 0.68 (0.58, 0.80) | 0.54 (0.49, 0.59) |
| 2021-2022 | 1.37 (1.20, 1.58) | 1.09 (1.03, 1.16) |
| Education  (High school or less) | 1.0 | 1.0 |
| Associate or Bachelor | 1.03 (0.95, 1.10) | 1.03 (0.95, 1.10) |
| PhD or Master’s | 0.93 (0.85, 1.01) | 0.93 (0.85, 1.01) |
| Military Status  (Active duty) | 1.0 | 1.0 |
| Retired military | 0.86 (0.77, 0.97) | 0.88 (0.78, 0.99) |
| Dependent | 0.84 (0.76, 0.93) | 0.85 (0.77, 0.94) |
| History of chronic pulmonary disease | 1.29 (1.14, 1.46) | 1.30 (1.14, 1.48) |
| Healthcare  (No) | 1.0 | 1.0 |
| Yes | 1.10 (1.04, 1.17) | 1.10 (1.04, 1.17) |
| Percent surveillance response | 1.00 (1.00, 1.00) | 1.00 (1.00, 1.00) |
| Smoking Status = Cigarettes; User Status = E-cigarettes   1. e-cigarette data was not collected in the 2018-2019 season | | |

Supplemental Table 4. Multivariable model results considering influenza-like illness (ILI) severity measures (FLU-PRO score, duration, number of days with fever or limited activity) as the outcome. Separate models were run that included cigarette smoking status or e-cigarette use; both models also included age, body mass index (BMI), influenza season, education level, military status, health care worker, and percent response to weekly surveillance surveys. The results for cigarette smoking status and electronic cigarette use status are shown in the table.

| FLU-PRO Scores | Cigarette smoking status | | | E-cigarette smoking status | | |
| --- | --- | --- | --- | --- | --- | --- |
|  | Non-smoker | Current Smoker | Former Smoker | Non-user | Current User | Former User |
| Total score | Reference | 0.10  (-0.03, 0.23) | 0.00  (-0.07, 0.08) | Reference | 0.11  (-0.02, 0.23) | 0.07  (-0.07, 0.22) |
| Nose score | Reference | 0.04  (-0.16, 0.25) | 0.05  (-0.06, 0.17) | Reference | -0.06  (-0.26, 0.14) | 0.10  (-0.13, 0.33) |
| Throat score | Reference | -0.06  (-0.28, 0.16) | -0.03  (-0.15, 0.10) | Reference | 0.20  (-0.02, 0.41) | 0.11  (-0.14, 0.37) |
| Eyes score | Reference | 0.11  (-0.06, 0.28) | -0.00  (-0.10, 0.09) | Reference | 0.20  (0.03, 0.36) | -0.08  (-0.27, 0.12) |
| Respiratory score | Reference | 0.24  (0.07, 0.41) | 0.07  (-0.03, 0.16) | Reference | 0.15  (-0.01, 0.32) | 0.27  (0.08, 0.46) |
| Gastrointestinal score | Reference | 0.08  (-0.05, 0.21) | 0.01  (-0.07, 0.08) | Reference | 0.12  (-0.01, 0.25) | 0.05  (-0.10, 0.20) |
| Systemic score | Reference | 0.08  (-0.10, 0.26) | -0.01  (-0.11, 0.09) | Reference | 0.11  (-0.06, 0.29) | 0.00  (-0.20, 0.21) |
|  | | | | | | |
| Duration | Reference | 0.56  (-1.06, 2.18) | 1.23  (0.29, 2.17) | Reference | 0.58  (-0.89, 2.04) | 1.52  (-0.24, 3.28) |
| Fever Days | Reference | 0.14  (-0.47, 0.75) | 0.25  (-0.10, 0.61) | Reference | 0.78  (0.20, 1.35) | 0.46  (-0.21, 1.14) |
| Limited Activity | Reference | 0.48  (-0.70, 1.65) | 0.43  (-0.26, 1.11) | Reference | 1.14  (0.10, 2.18) | 1.11  (-0.12, 2.33) |

Supplemental Figure 1. Sensitivity analysis performed by separating current smokers into two groups (7+ or <7 cigarettes in the last week (50% in each group))

**
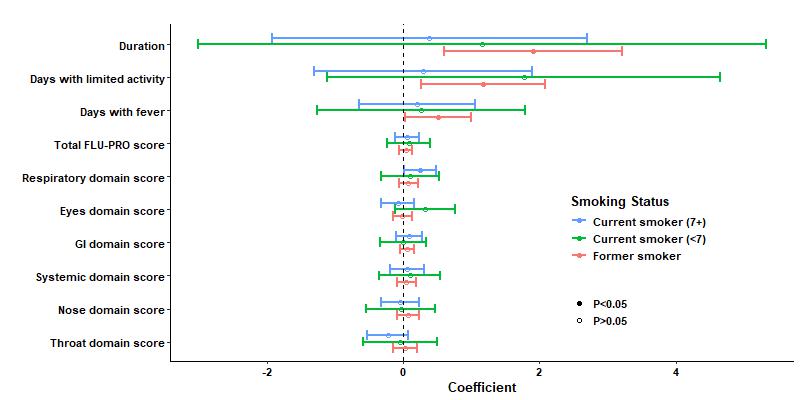
**

Supplemental Figure 2. Sensitivity analysis performed by separating current users into two groups 7+ or <7 times used a vaping device in the last week (64% <7 times, 36% 7+ times in each group))


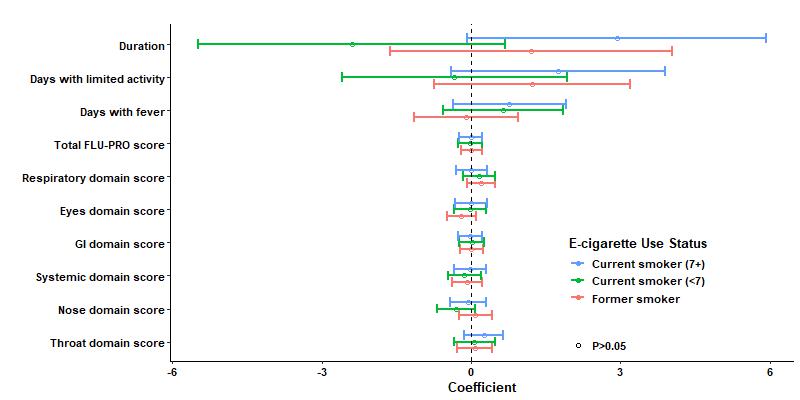


Supplemental Figure 3. Sensitivity analysis analyzing risk of influenza-like-illness using different categorizations of smoking status, grouped by color. Red: Any smoking, divided into 15+ years and <15 years of smoking; Dark blue: Separating current e-cigarette users into two groups 7+ or <7 times used a vaping device in the last week (64% <7 times, 36% 7+ times in each group)); Green: separating current smokers into two groups (7+ or <7 cigarettes in the last week (50% in each group)); Light blue: Combined e-cigarette and cigarette model; Yellow: Both cigarette and e-cigarette user compared to non-users; Pink: Any reported smoking/using compared to no reported smoking/using.


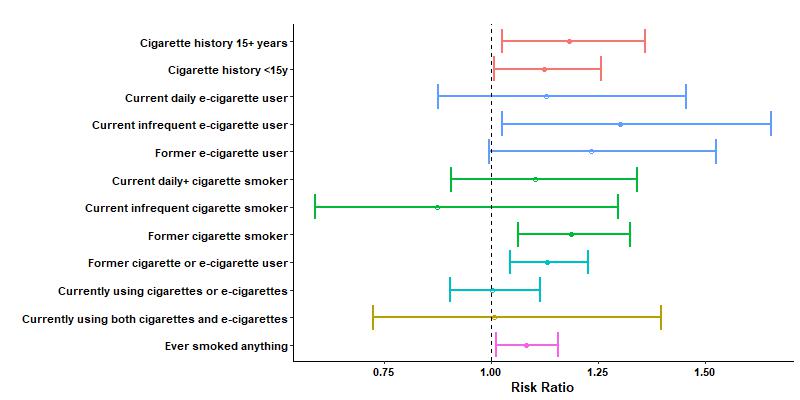

Supplement: Supplementary file 1 [file Supplementary_file_1.docx]
